# Beyond natural language: an ontology-based description of a new *Scarabaeus* dung beetle from Madagascar (Coleoptera, Scarabaeinae)

**DOI:** 10.3897/BDJ.12.e134364

**Published:** 2024-10-21

**Authors:** Giulio Montanaro, Sergei Tarasov

**Affiliations:** 1 Finnish Museum of Natural History, University of Helsinki, Helsinki, Finland Finnish Museum of Natural History, University of Helsinki Helsinki Finland

**Keywords:** dung beetles, computable phenotypes, taxonomy, semantic technologies, Phenoscript, nanopublications, FAIR data

## Abstract

**Background:**

The dung beetle genus *Scarabaeus* (Coleoptera, Scarabaeinae, Scarabaeini), predominantly found in the arid regions of the Old World, includes three endemic species inhabiting the dry ecosystems of western and southern Madagascar. These species are presumed to form a monophyletic clade nested within the African *Scarabaeus*.

Semantic modelling of phenotypes using ontologies represents a transformative approach to species description in biology, making phenotypic data FAIR and computable. The recently developed Phenoscript language enables the creation of semantic, computable species descriptions using a syntax akin to human natural language (NL). However, Phenoscript has not yet been tested as a tool for describing new taxa.

**New information:**

In this study, we test the utility of Phenoscript by describing a new species, *Scarabaeus (sensu lato) sakalava* sp. nov. from Madagascar. The initial description is composed directly in Phenoscript, replacing the traditional natural language format. This Phenoscript description is then translated into a human-readable form using the Phenospy tool for publication purposes. Additionally, the Phenoscript description is converted into an RDF graph, making it understandable by computers using semantic technologies.

*Scarabaeussakalava* sp. nov. is found in western central Madagascar and is closely related to *S.viettei* (Paulian, 1953) from north-western Madagascar. We provide an updated identification key and distribution map for all Malagasy *Scarabaeus* and discuss their systematic placement.

## Introduction

The tribe Scarabaeini comprises some of the most emblematic roller dung beetles, notably the "sacred scarabs" worshipped in Ancient Egypt (Cambefort et al. 1979). The group currently accounts for 181 described species divided into 11 genera, of which the most diverse is *Scarabaeus* Linnaeus, 1758 with 65 species (Daniel and Davis 2024, Schoolmeesters 2024). The members of this tribe are mostly open habitat specialists, occurring from moist savannahs to desert ecosystems of the Old World (Mostert and Scholtz 1986). Madagascar hosts three species all endemic to the country: *Scarabaeusradama* Fairmaire, 1895, *Scarabaeussevoistra* Alluaud, 1902 and *Scarabaeusviettei* (Paulian, 1953) (Deschodt et al. 2021). The first two species are restricted to the arid ericoid thickets and succulent woodlands in the south, while *S.viettei* occurs in dry deciduous forests in the northwest. No Scarabaeini are known from the more humid, forest-dominated regions of eastern and northern Madagascar (Rahagalala et al. 2009).

The most complete molecular phylogeny of the tribe showed that at least *S.viettei* and *S.radama* form a clade nested within a highly polyphyletic *Scarabaeus*, suggesting a single colonisation event of Madagascar from Africa (Sole et al. 2011). However, the flightless *S.sevoistra* possesses some morphologically derived traits that make its phylogenetic affinities still doubtful (Harrison et al. 2003, Harrison and Philips 2003, Sole et al. 2011).

During a recent survey of Malagasy dung beetles and by examining the collection of the Finnish Museum of Natural History (MZH), we found a new species of *Scarabaeus*, *S.sakalava* sp. nov., close to *S.viettei*. The new species is well differentiated from its sister both in terms of external and internal morphology and geographical distribution and can be separated from all other Malagasy species using the identification key provided below.

Species descriptions of *S.sakalava* and *S.viettei* are written using the semantic language Phenoscript. This language allows to write computer-parsable phenotypic statements that are directly linked to precisely defined terminology from biological ontologies (Mikó et al. 2021, Tarasov 2023, Montanaro et al. 2024a). This is the first publication in which a new species is described using a semantic language from scratch, which is then converted into traditional natural language version to facilitate readability and into an RDF graph to make it automatically queriable.

We discuss the utility of this semantic approach in creating taxonomic descriptions that are both human- and computer-readable, combining the benefits of semantic technologies with the obvious necessity of keeping descriptions intelligible to taxonomists. Additionally, we use nanopublications to communicate the establishment of the new species name and information about species-habitat association. Nanopubs represent promising tools to make taxonomic information FAIR – Findable, Accessible, Interoperable, Reusable (Montanaro et al. 2024a, Rossini et al. 2024).

Lastly, we discuss the evidence for the monophyly of Malagasy *Scarabaeus* and their systematic placement within Scarabaeini, as well as point out the need of a re-evaluation of the tribe's current supraspecific classification.

## Materials and methods

### Material and morphological examination

We studied nine specimens of *Scarabaeussakalava* sp. nov. collected using various baits (human or cattle dung, rotten fish). All specimens are dry preserved, except for three paratypes which are in 96% ethanol.

Additionally, we examined the holotype and 31 topotypic specimens of the closely-allied *Scarabaeusviettei* (Paulian, 1953), as well as non-type specimens of *Scarabaeusradama* Fairmaire, 1895. Morphological information about *Scarabaeussevoistra* was sourced from relevant literature (Alluaud 1902, Paulian and Lebis 1960, Mostert and Holm 1982, Harrison et al. 2003, Deschodt et al. 2021). Morphological terminology and protocols follow Tarasov and Génier (2015), Montanaro et al. (2024a) and Montanaro et al. (2024b). Specimens were examined under a Leica S9D stereomicroscope. Photographs were taken with a Canon MP-E 65 mm, f/2.8, 1–5× macro lens mounted on a Canon EOS 5D camera, stacked using the Stackshot (Cognisys Inc.) automated system and edited in Adobe Photoshop.

Initialisms of the collections where the material is preserved are the following:

- MNHN – Muséum national d'Histoire naturelle, Paris, France;
- MZH – Finnish Museum of Natural History, Helsinki, Finland.

A distribution map of all Malagasy *Scarabaeus* was created using QGIS v. 3.30.0 and annotated with Madagascar ecoregions (Olson et al. 2004). Additional distributional data about *S.radama*, *S.sevoistra* and *S.viettei* were retrieved from GBIF (GBIF.org 2024), iNaturalist (iNaturalist 2024) and Paulian and Lebis (1960). Full data of all specimens included in the study are available in Suppl. material 1.

### Semantic taxonomic descriptions

In using Phenoscript, we follow the general workflow proposed for *Grebennikovius* dung beetles (see details in Montanaro et al. (2024a)). However, previously, the semantic descriptions were compiled by manually rewriting the initial NL descriptions using Phenoscript language. In this paper, the NL description was entirely replaced by the human-readable NL-like version automatically generated using Phenospy tools from the Phenoscript description (i.e. phs files). Other aspects of the descriptive workflow and terminological conventions are the same used by Montanaro et al. (2024a) and Montanaro et al. (2024b). Present descriptions required the creation of 25 new anatomical terms in the AISM ontology (0000413–0000437) (Girón et al. 2024).

### Terminology from ontologies

**Figure 1.**
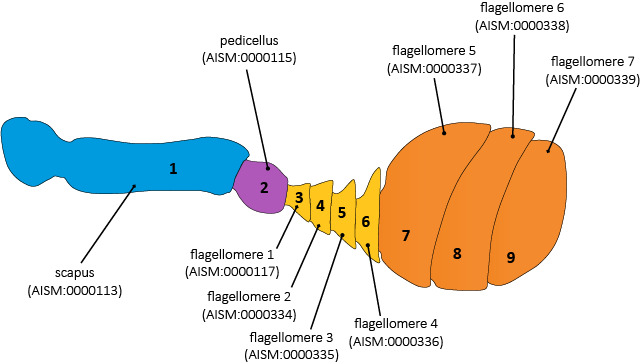
Typical 9-segmented dung beetle antenna explaining terminology for describing its parts. Numbers 1–9 indicate antennomeres. The first and second antennomeres are called scapus and pedicellus, respectively, while the following ones are called flagellomeres. In Scarabaeoidea and other coleopterans, a variable number of the distalmost flagellomeres are enlarged to form the antennal club (COLAO:0000267) (in orange). In Phenoscript, it is possible to describe which flagellomeres belong to the club by using this type of syntax: *aism-antenna_with_9_antennomeres > colao-antennal_club > (aism-flagellomere_5, aism-flagellomere_6, aism-flagellomere_7*).

The terminology used to describe antennomeres is illustrated in detail in Fig. 1. Notably, in different species of Scarabaeini meso- and metatibial spurs present various degrees of fusion with the tibia. A spur (AISM:0000040) which is not articulated anymore technically becomes a spine (AISM:0000527), which is the term we chose for describing these structures in our focal species. However, to keep track of the homology relationship between tibial spines and spurs, we described them as "*aism-cuticular_spine .ro-in_homology_relationship_with aism-metatibial_spur*" (see Mabee et al. (2020)).

### Nanopublications

The new taxon name statement and the association of *S.sakalava* and *S.viettei* with dry deciduous forest habitats are provided as nanopublications (Kuhn et al. 2013). Nanopubs were generated through nanodash, available through the Biodiversity Data Journal portal. For the new taxon name, a recently released, specific nanopub format was implemented.

## Taxon treatments

### Scarabaeus sakalava

Montanaro & Tarasov sp. nov.

955803D5-E4DB-5473-9285-CAA1D0680DA2

urn:lsid:zoobank.org:act:7AD8F87F-E7C1-4094-BD63-7662F167E9CB

#### Materials

- **Type status:** Holotype. **Occurrence:** catalogNumber: http://id.luomus.fi/GZ.15827; recordedBy: I. Hanski group; individualCount: 1; sex: male; lifeStage: adult; occurrenceID: 16BD880A-0D65-5B0E-909A-A3DDD3A4D1A8; **Taxon:** taxonID: urn:lsid:biosci.ohio-state.edu:osuc_names:275502; scientificName: Scarabaeussakalava; genus: Scarabaeus; specificEpithet: sakalava; scientificNameAuthorship: Montanaro & Tarasov; **Location:** country: Madagascar; stateProvince: Toliara; locality: Ambadira-Morondava; verbatimLocality: Ambadira-Mormdava; decimalLatitude: -19.8299; decimalLongitude: 44.6403; georeferenceRemarks: coordinates inferred from locality name; **Identification:** identifiedBy: Giulio Montanaro; dateIdentified: 2023; **Event:** samplingProtocol: cattle dung; eventDate: 12-2006; **Record Level:** institutionID: http://grbio.org/cool/2vmj-fp0v; institutionCode: MZH; basisOfRecord: PreservedSpecimen
- **Type status:** Paratype. **Occurrence:** catalogNumber: http://id.luomus.fi/GZ.15828; recordedBy: I. Hanski group; individualCount: 1; sex: female; lifeStage: adult; occurrenceID: 54A6436B-20BF-5615-9B08-048D138D820E; **Taxon:** taxonID: urn:lsid:biosci.ohio-state.edu:osuc_names:275502; scientificName: Scarabaeussakalava; genus: Scarabaeus; specificEpithet: sakalava; scientificNameAuthorship: Montanaro & Tarasov; **Location:** country: Madagascar; stateProvince: Toliara; locality: Ambadira-Morondava; verbatimLocality: Ambadira-Mormdava; decimalLatitude: -19.8299; decimalLongitude: 44.6403; georeferenceRemarks: coordinates inferred from locality name; **Identification:** identifiedBy: Giulio Montanaro; dateIdentified: 2023; **Event:** samplingProtocol: cattle dung; eventDate: 12-2006; **Record Level:** institutionID: http://grbio.org/cool/2vmj-fp0v; institutionCode: MZH; basisOfRecord: PreservedSpecimen
- **Type status:** Paratype. **Occurrence:** catalogNumber: http://id.luomus.fi/GZ.15818; recordedBy: Ilkka Hanski; individualCount: 1; sex: male; lifeStage: adult; occurrenceID: 341C47FD-A05B-53A0-99AB-30725CE58997; **Taxon:** taxonID: urn:lsid:biosci.ohio-state.edu:osuc_names:275502; scientificName: Scarabaeussakalava; genus: Scarabaeus; specificEpithet: sakalava; scientificNameAuthorship: Montanaro & Tarasov; **Location:** country: Madagascar; stateProvince: Toliara; locality: Marofandilia; decimalLatitude: -20.1327; decimalLongitude: 44.5503; georeferenceRemarks: coordinates inferred from locality name; **Identification:** identifiedBy: Giulio Montanaro; dateIdentified: 2023; **Event:** samplingProtocol: fish baited trap; eventDate: 04-2004; habitat: dry deciduous forest corridor; **Record Level:** institutionID: http://grbio.org/cool/2vmj-fp0v; institutionCode: MZH; basisOfRecord: PreservedSpecimen
- **Type status:** Paratype. **Occurrence:** catalogNumber: http://id.luomus.fi/GZ.15817; recordedBy: Ilkka Hanski; individualCount: 1; sex: male; lifeStage: adult; occurrenceID: F6002F94-267E-5CBB-802A-F519E4BEC873; **Taxon:** taxonID: urn:lsid:biosci.ohio-state.edu:osuc_names:275502; scientificName: Scarabaeussakalava; genus: Scarabaeus; specificEpithet: sakalava; scientificNameAuthorship: Montanaro & Tarasov; **Location:** country: Madagascar; stateProvince: Toliara; locality: Marofandilia; decimalLatitude: -20.1327; decimalLongitude: 44.5503; georeferenceRemarks: coordinates inferred from locality name; **Identification:** identifiedBy: Giulio Montanaro; dateIdentified: 2023; **Event:** samplingProtocol: fish baited trap; eventDate: 04-2004; habitat: dry deciduous forest corridor; **Record Level:** institutionID: http://grbio.org/cool/2vmj-fp0v; institutionCode: MZH; basisOfRecord: PreservedSpecimen
- **Type status:** Paratype. **Occurrence:** catalogNumber: http://id.luomus.fi/GZ.15816; recordedBy: Ilkka Hanski; individualCount: 1; sex: male; lifeStage: adult; occurrenceID: 7722D749-3E83-50B3-BB60-38B86F7F2477; **Taxon:** taxonID: urn:lsid:biosci.ohio-state.edu:osuc_names:275502; scientificName: Scarabaeussakalava; genus: Scarabaeus; specificEpithet: sakalava; scientificNameAuthorship: Montanaro & Tarasov; **Location:** country: Madagascar; stateProvince: Toliara; locality: Marofandilia; decimalLatitude: -20.1327; decimalLongitude: 44.5503; georeferenceRemarks: coordinates inferred from locality name; **Identification:** identifiedBy: Giulio Montanaro; dateIdentified: 2023; **Event:** samplingProtocol: fish baited trap; eventDate: 04-2004; habitat: dry deciduous forest corridor; **Record Level:** institutionID: http://grbio.org/cool/2vmj-fp0v; institutionCode: MZH; basisOfRecord: PreservedSpecimen
- **Type status:** Paratype. **Occurrence:** catalogNumber: http://id.luomus.fi/GZ.57485; recordedBy: Sergei Tarasov; individualCount: 1; sex: female; lifeStage: adult; occurrenceID: 2E216A5E-330D-5C21-9711-BB32E3B096D8; **Taxon:** scientificName: Scarabaeussakalava; genus: Scarabaeus; specificEpithet: sakalava; scientificNameAuthorship: Montanaro & Tarasov; **Location:** country: Madagascar; stateProvince: Toliara; locality: Morondava, Kirindy station; verbatimElevation: 71 m; verbatimCoordinates: -20.066805, 44.657255; decimalLatitude: -20.066805; decimalLongitude: 44.657255; georeferenceProtocol: GPS; **Identification:** identifiedBy: Giulio Montanaro; dateIdentified: 2023; **Event:** eventID: MG22-29a; samplingProtocol: human dung traps; eventDate: 03/07-03-2022; habitat: dry forest; **Record Level:** institutionID: http://grbio.org/cool/2vmj-fp0v; institutionCode: MZH; basisOfRecord: PreservedSpecimen
- **Type status:** Paratype. **Occurrence:** catalogNumber: http://id.luomus.fi/GZ.57486; recordedBy: Sergei Tarasov; individualCount: 1; sex: male; lifeStage: adult; occurrenceID: 055DDC5A-D731-5DB6-9A9C-B4359C2E84C5; **Taxon:** scientificName: Scarabaeussakalava; genus: Scarabaeus; specificEpithet: sakalava; scientificNameAuthorship: Montanaro & Tarasov; **Location:** country: Madagascar; stateProvince: Toliara; locality: Morondava, Kirindy station; verbatimElevation: 71 m; verbatimCoordinates: -20.066805, 44.657255; decimalLatitude: -20.066805; decimalLongitude: 44.657255; georeferenceProtocol: GPS; **Identification:** identifiedBy: Giulio Montanaro; dateIdentified: 2023; **Event:** eventID: MG22-29a; samplingProtocol: human dung traps; eventDate: 03/07-03-2022; habitat: dry forest; **Record Level:** institutionID: http://grbio.org/cool/2vmj-fp0v; institutionCode: MZH; basisOfRecord: PreservedSpecimen
- **Type status:** Paratype. **Occurrence:** catalogNumber: http://id.luomus.fi/GZ.57487; recordedBy: Sergei Tarasov; individualCount: 1; sex: female; lifeStage: adult; occurrenceID: 64E02FAA-3180-51C1-9ACC-C9E42D0C3020; **Taxon:** scientificName: Scarabaeussakalava; genus: Scarabaeus; specificEpithet: sakalava; scientificNameAuthorship: Montanaro & Tarasov; **Location:** country: Madagascar; stateProvince: Toliara; locality: Morondava, Kirindy station; verbatimElevation: 71 m; verbatimCoordinates: -20.066805, 44.657255; decimalLatitude: -20.066805; decimalLongitude: 44.657255; georeferenceProtocol: GPS; **Identification:** identifiedBy: Giulio Montanaro; dateIdentified: 2023; **Event:** eventID: MG22-29a; samplingProtocol: human dung traps; eventDate: 03/07-03-2022; habitat: dry forest; **Record Level:** institutionID: http://grbio.org/cool/2vmj-fp0v; institutionCode: MZH; basisOfRecord: PreservedSpecimen
- **Type status:** Paratype. **Occurrence:** catalogNumber: http://id.luomus.fi/GZ.57488; recordedBy: Sergei Tarasov; individualCount: 1; sex: female; lifeStage: adult; occurrenceID: 42053262-8E9F-5379-AD6C-6A6A57AA3507; **Taxon:** scientificName: Scarabaeussakalava; genus: Scarabaeus; specificEpithet: sakalava; scientificNameAuthorship: Montanaro & Tarasov; **Location:** country: Madagascar; stateProvince: Toliara; locality: Morondava, Kirindy station; verbatimElevation: 71 m; verbatimCoordinates: -20.066805, 44.657255; decimalLatitude: -20.066805; decimalLongitude: 44.657255; georeferenceProtocol: GPS; **Identification:** identifiedBy: Giulio Montanaro; dateIdentified: 2023; **Event:** eventID: MG22-29a; samplingProtocol: human dung traps; eventDate: 03/07-03-2022; habitat: dry forest; **Record Level:** institutionID: http://grbio.org/cool/2vmj-fp0v; institutionCode: MZH; basisOfRecord: PreservedSpecimen

#### Description

Fig. 2a, b

- male organism Catalog Number http://id.luomus.fi/GZ.15827
- male organism has role in modeling TU denotes species
  - species Parent Name Usage ID https://www.gbif.org/species/9074838
  - species Taxon ID http://zoobank.org/7AD8F87F-E7C1-4094-BD63-7662F167E9CB

- male organism: ovate;
- male organism, chitin-based cuticle
  - chitin-based cuticle, lateral side encircles cuticular seta: red brown;
  - chitin-based cuticle, ventral side encircles cuticular seta: red brown;
  - chitin-based cuticle: black;
  - chitin-based cuticle: glistening;
- male organism, antenna with 9 antennomeres
  - antenna with 9 antennomeres, antennal club
    - antennal club, flagellomere 5: present;
    - antennal club, flagellomere 6: present;
    - antennal club, flagellomere 7: present;
  - antenna with 9 antennomeres: red brown;
- male organism, insect leg encircles cuticular seta: red brown;
- male organism, genal margin
  - genal margin, posterior region: right angle to;
  - genal margin, anterior region
    - anterior region: protruding;
    - anterior region: acute angle to;
- male organism, head margin at genoclypeal sulcus: notched;
- male organism, lateral clypeal tooth 1: acute angle to;
- male organism, lateral clypeal tooth 2: acute angle to;
- male organism, clypeal margin between clypeal teeth 1
  - clypeal margin between clypeal teeth 1: acute angle to;
  - clypeal margin between clypeal teeth 1: notched;
- male organism, clypeal margin between clypeal teeth 1 and 2
  - clypeal margin between clypeal teeth 1 and 2: acute angle to;
  - clypeal margin between clypeal teeth 1 and 2: notched;
- male organism, frons
  - frons, cuticular carina
    - cuticular carina: bilaterally paired;
    - cuticular carina: transverse orientation;
    - cuticular carina: decreased length;
  - frons: protruding;
- male organism, vertex, granulated cuticle: present;
- male organism, gena
  - gena, medial region, granulated cuticle: present;
  - gena, lateral region: wrinkled;
- male organism, clypeus, anterior region: wrinkled;
- male organism, glossa: present;
- male organism, epipharynx: present;
- male organism, insect maxilla: present;
- male organism, maxillary palpus with 4 palpomeres: present;
- male organism, labial palpus with 3 palpomeres: present;
- male organism, pronotum
  - pronotum, dorsal region: convex;
  - pronotum, lateral margin: curved;
  - pronotum, posterior margin: curved;
  - pronotum, row of punctures coincident with posterior margin
  - pronotum: width larger than length of pronotum
- male organism, anterolateral pronotal angle
  - anterolateral pronotal angle: sharp;
  - anterolateral pronotal angle: lateral orientation;
- male organism, lateral pronotal carina
  - lateral pronotal carina, row of punctures: present;
  - lateral pronotal carina: serrated;
- male organism, pronotal disc, punctate cuticle, cuticular puncture: sparse;
- male organism, elytron with 9 striae
  - elytron with 9 striae, dorsal region: microreticulate;
  - elytron with 9 striae, elytral interstria
    - elytral interstria, punctate cuticle, cuticular puncture: sparse;
    - elytral interstria, granulated cuticle, cuticular granule
      - cuticular granule: decreased size;
      - cuticular granule: flattened;
- male organism, elytral interstria 8, proximal region, cuticular carina adjacent to elytral stria 7 of male organism
- male organism, elytral interstria 9, proximal region, cuticular carina adjacent to elytral stria 8 of male organism
- male organism, scutellar shield: concealed;
- male organism, hind wing
  - hind wing: present;
  - hind wing: normal;
- male organism, abdomen with 8 sternites: present;
- male organism, abdominal sternite VIII, cuticular groove
  - cuticular groove adjacent to postero-lateral margin of abdominal sternite VIII
  - cuticular groove: bilaterally paired;
- male organism, abdominal tergite VIII
  - abdominal tergite VIII, cuticular puncture
    - cuticular puncture: multiple;
    - cuticular puncture: sparse;
  - abdominal tergite VIII, anterior groove of tergite VIII: present;
  - abdominal tergite VIII, anterior carina of tergite VIII: present;
  - abdominal tergite VIII, distal border of tergite VIII, posterior region: increased length;
  - abdominal tergite VIII: convex;
  - abdominal tergite VIII: microreticulate;
- male organism, protarsus with 5 protarsomeres: present;
- male organism, mesotarsus with 5 mesotarsomeres: present;
- male organism, metatarsus with 5 metatarsomeres: present;
- male organism, protibia
  - protibia, dorsal protibial cuticular tooth 1: present;
  - protibia, dorsal protibial cuticular tooth 2: present;
  - protibia, dorsal protibial cuticular tooth 3: present;
  - protibia, dorsal protibial cuticular tooth 4: decreased size;
  - protibia, ventral margin: serrated;
  - protibia, ventral margin, proximal region: serrated;
  - protibia: curved;
- male organism, dorsal protibial margin between protibial teeth 1 and 2: scalloped;
- male organism, dorsal protibial margin between protibial teeth 2 and 3: scalloped;
- male organism, dorsal protibial margin between protibial teeth 3 and 4: scalloped;
- male organism, profemur, antero-ventral margin, distal region, cuticular spine: flattened;
- male organism, mesotibia
  - mesotibia, distal region, cuticular spine in homology relationship with mesotibial spur;
  - mesotibia, dorsal region, proximal region, mesotibial carina: length larger than length of mesotibial carina of medial region of dorsal region
- male organism, metatibia
  - metatibia, distal region, cuticular spine in homology relationship with metatibial spur;
  - metatibia, dorsal region, metatibial carina: length similar in magnitude relative to length of metatibial carina of dorsal region of metatibia
- male organism, mesofemur
  - mesofemur, anterior region, simple setigerous cuticular puncture: multiple;
  - mesofemur, ventral margin, simple setigerous cuticular puncture: multiple;
  - mesofemur, dorsal margin, distal region, simple setigerous cuticular puncture: multiple;
- male organism, mesotibial posteroventral carina, row of punctures, simple setigerous cuticular puncture: present;
- male organism, mesotibial anteroventral carina, row of punctures, simple setigerous cuticular puncture: present;
- male organism, mesotibial anterodorsal carina, row of punctures, simple setigerous cuticular puncture: present;
- male organism, mesotibial posterodorsal carina, row of punctures, simple setigerous cuticular puncture: present;
- male organism, metafemur
  - metafemur, anterior region, row of punctures, simple setigerous cuticular puncture: present;
  - metafemur, dorsal margin, simple setigerous cuticular puncture: multiple;
  - metafemur, ventral margin, proximal region, simple setigerous cuticular puncture: multiple;
- male organism, metatibial posteroventral carina, row of punctures
  - row of punctures, simple setigerous cuticular puncture: present;
  - row of punctures, distal region, simple setigerous cuticular puncture: amount larger than amount of simple setigerous cuticular puncture of proximal region of row of punctures
- male organism, metatibial anteroventral carina, row of punctures, simple setigerous cuticular puncture: present;
- male organism, metatibial anterodorsal carina, row of punctures, simple setigerous cuticular puncture: present;
- male organism, metatibial posterodorsal carina, row of punctures, simple setigerous cuticular puncture: present;
- male organism, parameres
  - parameres, dorsal margin: concave;
  - parameres, ventral margin: obtuse;
  - parameres, distal region
    - distal region, ventral margin: concave;
    - distal region, dorsal margin: straight angle to;
    - distal region: dorso-ventrally flattened;
  - parameres: asymmetrical;
- male organism, left paramere, ventral margin, cuticular protrusion: present;
- male organism, frontolateral peripheral endophallite
  - frontolateral peripheral endophallite, distal region: notched;
  - frontolateral peripheral endophallite: flattened;
  - frontolateral peripheral endophallite: elongated;
- male organism, axial endophallite: present;
- male organism, subaxial endophallite: present;
- male organism, superior right peripheral endophallite, lateral region: ring shaped;
- male organism, raspula, cuticular spine
  - cuticular spine: multiple;
  - cuticular spine: elongated;
- male organism, posterior longitudinal hypomeral carina: absent;
- male organism, anterior hypomeral carina: absent;
- male organism, lamella copulatrix: absent;
- male organism, length = 23.0, unit: millimeter;

##### Sexual dimorphism

Females differ from males by the following external characters: i) absence of head frontal carinae, which are replaced by a medial tubercle sinuate dorsally; ii) ventral and anterior margins of protibiae not serrated; iii) setae of posteroventral carina uniformly distributed, without denser rows distally; iv) abdominal tergite VIII shorter and 7^th^ abdominal sternite longer.

##### Variation

Body length ranges from 18.0 to 23.5 mm. The spines on ventral and anterior margins of male protibiae are more developed in larger individuals.

##### Holotype labelling

The holotype bears the following labels (slashes separate different lines on the same label). 1^st^ label, white cardboard printed in black: "MADAGASCAR / Ambadira-Mormdava / Dec. 2006 / Cattle dung / I. Hanski-group leg.". 2^nd^ label, white cardboard printed in black, including a QR-code: "http://id.luomus.fi/ / GZ.15827 / XII.2006". 3^rd^ label, red cardboard printed in black: "*Scarabaeus* (*sensu lato*) / *sakalava* sp. nov. / HOLOTYPE / Montanaro & Tarasov det. 2024"

#### Diagnosis

*Scarabaeussakalava* sp. nov. (Fig. 2a, b) is very similar to *Scarabaeusviettei* (Paulian, 1953) (Fig. 2c, d), from which it can be distinguished by the tip of the anterolateral pronotal angles, projecting laterally in *S.sakalava* (Fig. 3a) and obliquely forward in *S.viettei* (Fig. 3b). Females can be readily separated by the shape of the cephalic tubercle, sinuated medially in the new species (Fig. 3c) and conical in *S.viettei* (Fig. 3d). Males can be distinguished by the shape of parameres, which are shorter and whose apex is more strongly bent ventrally in *S.sakalava* (Fig. 4a) than in the other species (Fig. 4b) and by the shape of the protibia, less slender and with the ventral margin more dilated in the new species (Fig. 3e) than in *S.viettei* (Fig. 3f). Lastly, the two species can be immediately distinguished from *S.radama* by the much shallower and sparse integument punctuation and from *S.sevoistra* by the normally-shaped elytra and hind wings (modified due to flightlessness in the latter species).

#### Etymology

The new species is named after the Sakalava people inhabiting western Madagascar, where the species occurs. The name *sakalava* probably means "long ravines/valleys", denoting the relatively flat landscapes of western Madagascar. The Sakalavas descend from a mix of Austronesian and Bantu people and founded kingdoms that flourished in the west of the country, especially during the 18^th^ century (Wikipedia 2024). They primarily rely on pastoralism for their livelihood, therefore providing an arguably significant part of the sustenance consumed by new species. The epithet is a noun in apposition.

#### Distribution

**Figure 5.**
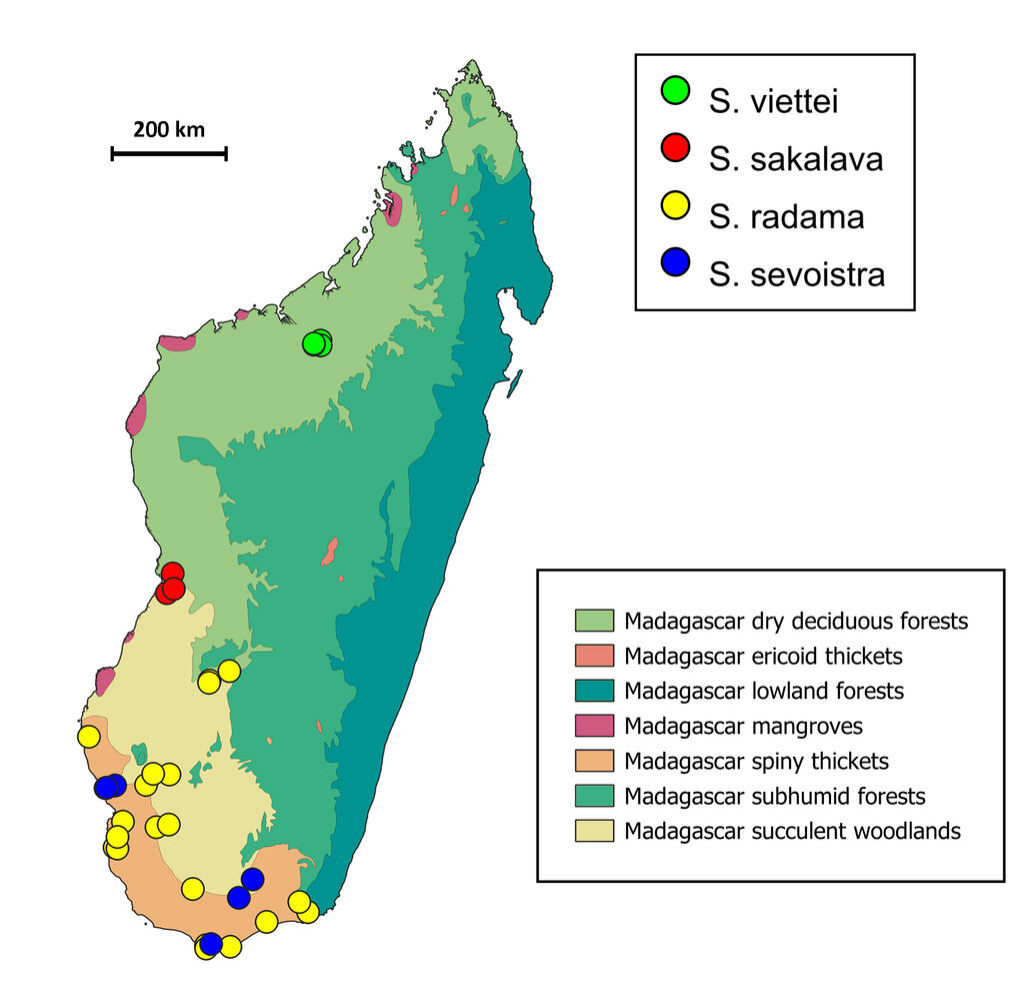
Distribution map of Malagasy *Scarabaeus* annotated with ecoregions.

*S.sakalava* is found in a restricted area in central western Madagascar, in Toliara Province (Fig. 5).

#### Ecology

*S.sakalava* inhabits dry deciduous forests, a relatively unusual habitat specialisation for *Scarabaeus* species, which are, for the greatest part, found in open habitats (Mostert and Scholtz 1986). It is attracted to human and cattle dung and to rotten fish. These ecological aspects are very similar to those of its sister *S.viettei* (Rahagalala et al. 2009), which seems to be its vicariant in north-western Madagascar (Ankarafantsika Forest).

#### Conservation

The known distribution of the species falls within the Menabe-Antimena Protected Area, which should, for now, guarantee the protection of this taxon.

### Scarabaeus viettei

(Paulian, 1953)

1801FAE3-8208-5C11-B6C3-8608244F3C98

Madateuchus viettei : Paulian (1953): 25; Paulian and Lebis (1960): 12; Harrison et al. (2003): 349; Ziani and Gudenzi (2012): 139;
Scarabaeus viettei : Mostert and Scholtz (1986): 11; Zídek and Pokorný (2004): 10; Rahagalala et al. (2009): 71; Sole et al. (2011): 31; Deschodt et al. (2021): 4.

#### Materials

- **Type status:** Other material. **Occurrence:** recordedBy: A. Peyrieras; individualCount: 5; sex: male; lifeStage: adult; occurrenceID: A16A41AD-FA81-5488-BC2D-3F53D3C45779; **Taxon:** taxonID: urn:lsid:biosci.ohio-state.edu:osuc_names:275502; scientificName: Scarabaeusviettei; scientificNameAuthorship: (Paulian, 1953); **Location:** country: Madagascar; stateProvince: Mahajanga; locality: Ankarafantsika, Ampijoroa Lake; verbatimLocality: MADAGASCAR OUEST, ANPIJORO, Ankarafantsy Lac; decimalLatitude: -16.312; decimalLongitude: 46.816; georeferenceProtocol: GPS; georeferenceRemarks: coordinates inferred from locality name; **Identification:** identifiedBy: Giulio Montanaro; dateIdentified: 2023; **Event:** eventDate: 01-1976; **Record Level:** institutionID: http://biocol.org/urn:lsid:biocol.org:col:34988; institutionCode: MNHN; basisOfRecord: PreservedSpecimen
- **Type status:** Other material. **Occurrence:** recordedBy: A. Peyrieras; individualCount: 13; sex: female; lifeStage: adult; occurrenceID: 747D9F5E-BB05-5CAE-AD82-3A5552796FF8; **Taxon:** taxonID: urn:lsid:biosci.ohio-state.edu:osuc_names:275502; scientificName: Scarabaeusviettei; scientificNameAuthorship: (Paulian, 1953); **Location:** country: Madagascar; stateProvince: Mahajanga; locality: Ankarafantsika, Ampijoroa Lake; verbatimLocality: MADAGASCAR OUEST, ANPIJORO, Ankarafantsy Lac; decimalLatitude: -16.312; decimalLongitude: 46.816; georeferenceProtocol: GPS; georeferenceRemarks: coordinates inferred from locality name; **Identification:** identifiedBy: Giulio Montanaro; dateIdentified: 2023; **Event:** eventDate: 01-1976; **Record Level:** institutionID: http://biocol.org/urn:lsid:biocol.org:col:34988; institutionCode: MNHN; basisOfRecord: PreservedSpecimen
- **Type status:** Other material. **Occurrence:** individualCount: 1; sex: male; lifeStage: adult; occurrenceID: CEC30914-A927-5C1B-8E73-FA8C6D7F7DF0; **Taxon:** scientificName: Scarabaeusviettei; scientificNameAuthorship: (Paulian, 1953); **Location:** country: Madagascar; stateProvince: Mahajanga; locality: Ankarafantsika forest, Raharizonina; verbatimLocality: Madagascar Nord-Ouest, det. Majunga, foręt Ankarafantsika 1200m, Raharizonina; decimalLatitude: -16.25; decimalLongitude: 46.917; georeferenceProtocol: GPS; georeferenceRemarks: coordinates inferred from locality name; **Identification:** identifiedBy: Giulio Montanaro; dateIdentified: 2023; **Event:** eventDate: 12-1959; **Record Level:** institutionID: http://biocol.org/urn:lsid:biocol.org:col:34988; institutionCode: MNHN; basisOfRecord: PreservedSpecimen
- **Type status:** Other material. **Occurrence:** recordedBy: Peyrieras; individualCount: 1; sex: male; lifeStage: adult; occurrenceID: 8A2EF327-5052-5B91-84A7-90519633C114; **Taxon:** scientificName: Scarabaeusviettei; scientificNameAuthorship: (Paulian, 1953); **Location:** country: Madagascar; stateProvince: Mahajanga; locality: Ankarafantsika, Ampijoroa Lake; verbatimLocality: Ankarafantsy, Lac Ampijoroa, Peyrieras-III; decimalLatitude: -16.312; decimalLongitude: 46.816; georeferenceProtocol: GPS; georeferenceRemarks: coordinates inferred from locality name; **Identification:** identifiedBy: Giulio Montanaro; dateIdentified: 2023; **Event:** eventDate: Peyrieras-III; **Record Level:** institutionID: http://biocol.org/urn:lsid:biocol.org:col:34988; institutionCode: MNHN; basisOfRecord: PreservedSpecimen
- **Type status:** Other material. **Occurrence:** recordedBy: Peyrieras; individualCount: 1; sex: female; lifeStage: adult; occurrenceID: EA2E4B39-6E96-56A2-B92E-9DB6A21E9BD3; **Taxon:** scientificName: Scarabaeusviettei; scientificNameAuthorship: (Paulian, 1953); **Location:** country: Madagascar; stateProvince: Mahajanga; locality: Ankarafantsika, Ampijoroa Lake; verbatimLocality: Ankarafantsy, Lac Ampijoroa, Peyrieras-III; decimalLatitude: -16.312; decimalLongitude: 46.816; georeferenceProtocol: GPS; georeferenceRemarks: coordinates inferred from locality name; **Identification:** identifiedBy: Giulio Montanaro; dateIdentified: 2023; **Event:** eventDate: Peyrieras-III; **Record Level:** institutionID: http://biocol.org/urn:lsid:biocol.org:col:34988; institutionCode: MNHN; basisOfRecord: PreservedSpecimen
- **Type status:** Other material. **Occurrence:** individualCount: 2; sex: male; lifeStage: adult; occurrenceID: 77C5EDEF-10AE-5357-B812-74C45749A48C; **Taxon:** scientificName: Scarabaeusviettei; scientificNameAuthorship: (Paulian, 1953); **Location:** country: Madagascar; stateProvince: Mahajanga; locality: Ampijoroa; verbatimLocality: Ampijoroa, (XI/IX); decimalLatitude: -16.312; decimalLongitude: 46.816; georeferenceProtocol: GPS; georeferenceRemarks: coordinates inferred from locality name; **Identification:** identifiedBy: Giulio Montanaro; dateIdentified: 2023; **Record Level:** institutionID: http://biocol.org/urn:lsid:biocol.org:col:34988; institutionCode: MNHN; basisOfRecord: PreservedSpecimen
- **Type status:** Other material. **Occurrence:** catalogNumber: http://id.luomus.fi/GZ.15819; recordedBy: Hanski group; individualCount: 1; sex: male; lifeStage: adult; occurrenceID: E37678DE-4775-5166-A905-E7C661B168DA; **Taxon:** scientificName: Scarabaeusviettei; scientificNameAuthorship: (Paulian, 1953); **Location:** country: Madagascar; stateProvince: Mahajanga; locality: Ampondrabe, near Ankarafantsika; verbatimLocality: Madagascar, Ampondrabe, near Ankarafantsika; verbatimElevation: 256m; verbatimCoordinates: 16°19'28"S, 046°55'09"E; decimalLatitude: -16.3244; decimalLongitude: 46.9191; georeferenceProtocol: label; **Identification:** identifiedBy: Giulio Montanaro; dateIdentified: 2023; **Event:** samplingProtocol: fish bait trap; eventDate: 23-11-2006; **Record Level:** institutionID: http://grbio.org/cool/2vmj-fp0v; institutionCode: MZH; basisOfRecord: PreservedSpecimen
- **Type status:** Other material. **Occurrence:** catalogNumber: http://id.luomus.fi/GZ.15820; recordedBy: Hanski group; individualCount: 1; sex: male; lifeStage: adult; occurrenceID: 6FFA3662-FB0D-54EC-A251-716F138B6B3B; **Taxon:** scientificName: Scarabaeusviettei; scientificNameAuthorship: (Paulian, 1953); **Location:** country: Madagascar; stateProvince: Mahajanga; locality: Ampondrabe, near Ankarafantsika; verbatimLocality: Madagascar, Ampondrabe, near Ankarafantsika; verbatimElevation: 256m; verbatimCoordinates: 16°19'28"S, 046°55'09"E; decimalLatitude: -16.3244; decimalLongitude: 46.9191; georeferenceProtocol: label; **Identification:** identifiedBy: Giulio Montanaro; dateIdentified: 2023; **Event:** samplingProtocol: fish bait trap; eventDate: 23-11-2006; **Record Level:** institutionID: http://grbio.org/cool/2vmj-fp0v; institutionCode: MZH; basisOfRecord: PreservedSpecimen
- **Type status:** Other material. **Occurrence:** catalogNumber: http://id.luomus.fi/GZ.15821; recordedBy: Hanski group; individualCount: 1; sex: male; lifeStage: adult; occurrenceID: 112DB7C2-AE7A-507F-A804-910C523E4866; **Taxon:** scientificName: Scarabaeusviettei; scientificNameAuthorship: (Paulian, 1953); **Location:** country: Madagascar; stateProvince: Mahajanga; locality: Ampondrabe, near Ankarafantsika; verbatimLocality: Madagascar, Ampondrabe, near Ankarafantsika; verbatimElevation: 256m; verbatimCoordinates: 16°19'28"S, 046°55'09"E; decimalLatitude: -16.3244; decimalLongitude: 46.9191; georeferenceProtocol: label; **Identification:** identifiedBy: Giulio Montanaro; dateIdentified: 2023; **Event:** samplingProtocol: fish bait trap; eventDate: 23-11-2006; **Record Level:** institutionID: http://grbio.org/cool/2vmj-fp0v; institutionCode: MZH; basisOfRecord: PreservedSpecimen
- **Type status:** Other material. **Occurrence:** catalogNumber: http://id.luomus.fi/GZ.15822; recordedBy: Hanski group; individualCount: 1; sex: male; lifeStage: adult; occurrenceID: 03730B41-3119-567D-9F07-87A98D3C84EB; **Taxon:** scientificName: Scarabaeusviettei; scientificNameAuthorship: (Paulian, 1953); **Location:** country: Madagascar; stateProvince: Mahajanga; locality: Ampondrabe, near Ankarafantsika; verbatimLocality: Madagascar, Ampondrabe, near Ankarafantsika; verbatimElevation: 256 m; verbatimCoordinates: 16°19'28"S, 046°55'09"E; decimalLatitude: -16.3244; decimalLongitude: 46.9191; georeferenceProtocol: label; **Identification:** identifiedBy: Giulio Montanaro; dateIdentified: 2023; **Event:** samplingProtocol: fish bait trap; eventDate: 23-11-2006; **Record Level:** institutionID: http://grbio.org/cool/2vmj-fp0v; institutionCode: MZH; basisOfRecord: PreservedSpecimen
- **Type status:** Other material. **Occurrence:** catalogNumber: http://id.luomus.fi/GZ.15826; recordedBy: Hanski group; individualCount: 1; sex: male; lifeStage: adult; occurrenceID: 974DA766-B5B6-57C8-B8B6-FC11114F5DAA; **Taxon:** scientificName: Scarabaeusviettei; scientificNameAuthorship: (Paulian, 1953); **Location:** country: Madagascar; stateProvince: Mahajanga; locality: Ampondrabe, near Ankarafantsika; verbatimLocality: Madagascar, Ampondrabe, near Ankarafantsika; verbatimElevation: 256 m; verbatimCoordinates: 16°19'28"S, 046°55'09"E; decimalLatitude: -16.3244; decimalLongitude: 46.9191; georeferenceProtocol: label; **Identification:** identifiedBy: Giulio Montanaro; dateIdentified: 2023; **Event:** samplingProtocol: fish bait trap; eventDate: 23-11-2006; **Record Level:** institutionID: http://grbio.org/cool/2vmj-fp0v; institutionCode: MZH; basisOfRecord: PreservedSpecimen
- **Type status:** Other material. **Occurrence:** catalogNumber: http://id.luomus.fi/GZ.15823; recordedBy: Hanski group; individualCount: 1; sex: female; lifeStage: adult; occurrenceID: 208F3440-6AB2-58CB-AAF1-5F689F69A930; **Taxon:** scientificName: Scarabaeusviettei; scientificNameAuthorship: (Paulian, 1953); **Location:** country: Madagascar; stateProvince: Mahajanga; locality: Ampondrabe, near Ankarafantsika; verbatimLocality: Madagascar, Ampondrabe, near Ankarafantsika; verbatimElevation: 256 m; verbatimCoordinates: 16°19'28"S, 046°55'09"E; decimalLatitude: -16.3244; decimalLongitude: 46.9191; georeferenceProtocol: label; **Identification:** identifiedBy: Giulio Montanaro; dateIdentified: 2023; **Event:** samplingProtocol: fish bait trap; eventDate: 23-11-2006; **Record Level:** institutionID: http://grbio.org/cool/2vmj-fp0v; institutionCode: MZH; basisOfRecord: PreservedSpecimen
- **Type status:** Other material. **Occurrence:** catalogNumber: http://id.luomus.fi/GZ.15824; recordedBy: Hanski group; individualCount: 1; sex: female; lifeStage: adult; occurrenceID: 1E2D8366-406B-5E11-BE2D-CB31C1249099; **Taxon:** scientificName: Scarabaeusviettei; scientificNameAuthorship: (Paulian, 1953); **Location:** country: Madagascar; stateProvince: Mahajanga; locality: Ampondrabe, near Ankarafantsika; verbatimLocality: Madagascar, Ampondrabe, near Ankarafantsika; verbatimElevation: 256 m; verbatimCoordinates: 16°19'28"S, 046°55'09"E; decimalLatitude: -16.3244; decimalLongitude: 46.9191; georeferenceProtocol: label; **Identification:** identifiedBy: Giulio Montanaro; dateIdentified: 2023; **Event:** samplingProtocol: fish bait trap; eventDate: 23-11-2006; **Record Level:** institutionID: http://grbio.org/cool/2vmj-fp0v; institutionCode: MZH; basisOfRecord: PreservedSpecimen
- **Type status:** Other material. **Occurrence:** catalogNumber: http://id.luomus.fi/GZ.15825; recordedBy: Hanski group; individualCount: 1; sex: female; lifeStage: adult; occurrenceID: AD252859-E67C-5004-AC2B-AC8BB4E6BD2C; **Taxon:** scientificName: Scarabaeusviettei; scientificNameAuthorship: (Paulian, 1953); **Location:** country: Madagascar; stateProvince: Mahajanga; locality: Ampondrabe, near Ankarafantsika; verbatimLocality: Madagascar, Ampondrabe, near Ankarafantsika; verbatimElevation: 256 m; verbatimCoordinates: 16°19'28"S, 046°55'09"E; decimalLatitude: -16.3244; decimalLongitude: 46.9191; georeferenceProtocol: label; **Identification:** identifiedBy: Giulio Montanaro; dateIdentified: 2023; **Event:** samplingProtocol: fish bait trap; eventDate: 23-11-2006; **Record Level:** institutionID: http://grbio.org/cool/2vmj-fp0v; institutionCode: MZH; basisOfRecord: PreservedSpecimen

#### Description

Fig. 2c, d

- male organism Catalog Number http://id.luomus.fi/GZ.15821
- male organism has role in modeling TU denotes species
  - species Parent Name Usage ID https://www.gbif.org/species/9074838
  - species Taxon ID https://www.gbif.org/species/4997091

- male organism: ovate;
- male organism, chitin-based cuticle
  - chitin-based cuticle, lateral side encircles cuticular seta: red brown;
  - chitin-based cuticle, ventral side encircles cuticular seta: red brown;
  - chitin-based cuticle: black;
  - chitin-based cuticle: glistening;
- male organism, antenna with 9 antennomeres
  - antenna with 9 antennomeres, antennal club
    - antennal club, flagellomere 5: present;
    - antennal club, flagellomere 6: present;
    - antennal club, flagellomere 7: present;
  - antenna with 9 antennomeres: red brown;
- male organism, insect leg encircles cuticular seta: red brown;
- male organism, genal margin
  - genal margin, posterior region: right angle to;
  - genal margin, anterior region
    - anterior region: protruding;
    - anterior region: acute angle to;
- male organism, head margin at genoclypeal sulcus: notched;
- male organism, lateral clypeal tooth 1: acute angle to;
- male organism, lateral clypeal tooth 2: acute angle to;
- male organism, clypeal margin between clypeal teeth 1
  - clypeal margin between clypeal teeth 1: acute angle to;
  - clypeal margin between clypeal teeth 1: notched;
- male organism, clypeal margin between clypeal teeth 1 and 2
  - clypeal margin between clypeal teeth 1 and 2: acute angle to;
  - clypeal margin between clypeal teeth 1 and 2: notched;
- male organism, frons
  - frons, cuticular carina
    - cuticular carina: bilaterally paired;
    - cuticular carina: transverse orientation;
    - cuticular carina: decreased length;
  - frons: protruding;
- male organism, vertex, granulated cuticle: present;
- male organism, gena, dorsal region
  - dorsal region, medial region, granulated cuticle: present;
  - dorsal region, lateral region: wrinkled;
- male organism, clypeus, dorsal region, anterior region: wrinkled;
- male organism, glossa: present;
- male organism, epipharynx: present;
- male organism, insect maxilla: present;
- male organism, maxillary palpus with 4 palpomeres: present;
- male organism, labial palpus with 3 palpomeres: present;
- male organism, pronotum
  - pronotum, dorsal region: convex;
  - pronotum, lateral margin: curved;
  - pronotum, posterior margin: curved;
  - pronotum, row of punctures coincident with posterior margin
  - pronotum: width larger than length of pronotum
- male organism, anterolateral pronotal angle
  - anterolateral pronotal angle: sharp;
  - anterolateral pronotal angle: oblique orientation;
- male organism, lateral pronotal carina
  - lateral pronotal carina, row of punctures: present;
  - lateral pronotal carina: serrated;
- male organism, pronotal disc, punctate cuticle, cuticular puncture: sparse;
- male organism, elytron with 9 striae
  - elytron with 9 striae, dorsal region: microreticulate;
  - elytron with 9 striae, elytral interstria
    - elytral interstria, punctate cuticle, cuticular puncture: sparse;
    - elytral interstria, granulated cuticle, cuticular granule
      - cuticular granule: decreased size;
      - cuticular granule: flattened;
- male organism, elytral interstria 8, proximal region, cuticular carina adjacent to elytral stria 7 of male organism
- male organism, elytral interstria 9, proximal region, cuticular carina adjacent to elytral stria 8 of male organism
- male organism, scutellar shield: concealed;
- male organism, hind wing
  - hind wing: present;
  - hind wing: normal;
- male organism, abdomen with 8 sternites: present;
- male organism, abdominal sternite VIII, cuticular groove
  - cuticular groove adjacent to postero-lateral margin of abdominal sternite VIII
  - cuticular groove: bilaterally paired;
- male organism, abdominal tergite VIII
  - abdominal tergite VIII, cuticular puncture
    - cuticular puncture: multiple;
    - cuticular puncture: sparse;
  - abdominal tergite VIII, anterior groove of tergite VIII: present;
  - abdominal tergite VIII, anterior carina of tergite VIII: present;
  - abdominal tergite VIII, distal border of tergite VIII, posterior region: increased length;
  - abdominal tergite VIII: convex;
  - abdominal tergite VIII: microreticulate;
- male organism, protarsus with 5 protarsomeres: present;
- male organism, mesotarsus with 5 mesotarsomeres: present;
- male organism, metatarsus with 5 metatarsomeres: present;
- male organism, protibia
  - protibia, dorsal protibial cuticular tooth 1: present;
  - protibia, dorsal protibial cuticular tooth 2: present;
  - protibia, dorsal protibial cuticular tooth 3: present;
  - protibia, dorsal protibial cuticular tooth 4: decreased size;
  - protibia, ventral margin: serrated;
  - protibia, ventral margin, proximal region: serrated;
  - protibia: curved;
- male organism, dorsal protibial margin between protibial teeth 1 and 2: scalloped;
- male organism, dorsal protibial margin between protibial teeth 2 and 3: scalloped;
- male organism, dorsal protibial margin between protibial teeth 3 and 4: scalloped;
- male organism, profemur, antero-ventral margin, distal region, cuticular spine: flattened;
- male organism, mesotibia
  - mesotibia, distal region, cuticular spine in homology relationship with mesotibial spur;
  - mesotibia, dorsal region, proximal region, mesotibial carina: length larger than length of mesotibial carina of medial region of dorsal region
- male organism, metatibia
  - metatibia, distal region, cuticular spine in homology relationship with metatibial spur;
  - metatibia, dorsal region, metatibial carina: length similar in magnitude relative to length of metatibial carina of dorsal region of metatibia
- male organism, mesofemur
  - mesofemur, anterior region, simple setigerous cuticular puncture: multiple;
  - mesofemur, ventral margin, simple setigerous cuticular puncture: multiple;
  - mesofemur, dorsal margin, distal region, simple setigerous cuticular puncture: multiple;
- male organism, mesotibial posteroventral carina, row of punctures, simple setigerous cuticular puncture: present;
- male organism, mesotibial anteroventral carina, row of punctures, simple setigerous cuticular puncture: present;
- male organism, mesotibial anterodorsal carina, row of punctures, simple setigerous cuticular puncture: present;
- male organism, mesotibial posterodorsal carina, row of punctures, simple setigerous cuticular puncture: present;
- male organism, metafemur
  - metafemur, anterior region, row of punctures, simple setigerous cuticular puncture: present;
  - metafemur, dorsal margin, simple setigerous cuticular puncture: multiple;
  - metafemur, ventral margin, proximal region, simple setigerous cuticular puncture: multiple;
- male organism, metatibial posteroventral carina, row of punctures
  - row of punctures, simple setigerous cuticular puncture: present;
  - row of punctures, distal region, simple setigerous cuticular puncture: amount larger than amount of simple setigerous cuticular puncture of proximal region of row of punctures
- male organism, metatibial anteroventral carina, row of punctures, simple setigerous cuticular puncture: present;
- male organism, metatibial anterodorsal carina, row of punctures, simple setigerous cuticular puncture: present;
- male organism, metatibial posterodorsal carina, row of punctures, simple setigerous cuticular puncture: present;
- male organism, parameres
  - parameres, dorsal margin: concave;
  - parameres, ventral margin: obtuse;
  - parameres, distal region
    - distal region, ventral margin: concave;
    - distal region, dorsal margin: obtuse angle to;
    - distal region: dorso-ventrally flattened;
  - parameres: asymmetrical;
- male organism, left paramere, ventral margin, cuticular protrusion: present;
- male organism, frontolateral peripheral endophallite
  - frontolateral peripheral endophallite, distal region: notched;
  - frontolateral peripheral endophallite: flattened;
  - frontolateral peripheral endophallite: elongated;
- male organism, axial endophallite: present;
- male organism, subaxial endophallite: present;
- male organism, superior right peripheral endophallite, lateral region: ring shaped;
- male organism, raspula, cuticular spine
  - cuticular spine: multiple;
  - cuticular spine: elongated;
- male organism, posterior longitudinal hypomeral carina: absent;
- male organism, anterior hypomeral carina: absent;
- male organism, lamella copulatrix: absent;
- male organism, length = 21.5, unit: millimeter;

##### Sexual dimorphism

Females differ from males by the following external characters: i) absence of head frontal carinae, which are replaced by a conical medial tubercle; ii) ventral and anterior margins of protibiae not serrated; iii) setae of posteroventral carina uniformly distributed, without denser rows distally; iv) abdominal tergite VIII shorter and 7^th^ abdominal sternite longer.

##### Variation

Body size ranges from 21.5 to 23.0 mm (n=3).

#### Diagnosis

See diagnosis of *S.sakalava*.

#### Distribution

*S.viettei* seems to be a narrow endemic of Ankarafantsika Forest in north-eastern Madagascar, Boeny Region (see Fig. 5).

#### Ecology

Similarly to *S.sakalava* (see above), this species seems to be restricted to dry deciduous forest habitats. It was collected using cattle dung, human dung and rotten fish (Rahagalala et al. 2009).

#### Conservation

The known distribution of the species falls within the Ankarafantsika National Park, which should guarantee, for now, the protection of this species.

#### Etymology

The species was dedicated by Paulian to Pierre E. L. Viette (1921–2011), who collected the holotype. Viette was a French entomologist specialised in Lepidoptera, who conducted several expeditions to Madagascar and other islands in the Indian Ocean (Wikipedia 2021).

## Identification Keys

### Identification key to adult Malagasy *Scarabaeus*

**Table:** 

1	Pronotal punctuation strong, granulose and very dense (punctures separated by 1 puncture diameter or less). Colour metallic bronze to coppery	***S.radama***
–	Pronotal punctuation weaker, simple and much sparser (punctures separated by 5 diameters or more). Colour black	2
2	Wings reduced, elytra narrower than pronotum. Distal end of male protibia curved. Female head without tubercles	***S.sevoistra***
–	Wings fully developed, elytra as wide or slightly wider than pronotum. Distal end of male protibia straight. Female head with a frontal tubercle	3
3	Anterolateral angle of pronotum directed laterally (Fig. 3a). Female head tubercle sinuated medially (Fig. 3c). Ventral margin of male protibia expanded (Fig. 3e). Parameres less slender, apex forming a straight angle with dorso-distal margin in lateral view (Fig. 4a)	***S.sakalava***
–	Anterolateral angle of pronotum directed obliquely forward (Fig. 3b). Female head tubercle conical (Fig. 3d). Ventral margin of male protibia poorly expanded (Fig. 3f). Parameres more slender, apex creating an obtuse angle with dorso-distal margin in lateral view (Fig. 4b)	***S.viettei***

## Discussion

### Phenoscript: an efficient and sufficient tool to describe species from scratch

One of the challenges of modern taxonomy is to make the huge amount of phenotypic data easily available across various biological domains (Deans et al. 2012). The aim of semantic taxonomy is to make phenotypic data computable and FAIR, so that they can be easily mined and re-utilised using informatic tools and their usefulness does not end within taxonomic publications (Yoder et al. 2018). The use of semantic languages is a field under active development, which, so far, only a few taxonomists have explored to describe biodiversity (Balhoff et al. 2013, Mikó et al. 2021, Montanaro et al. 2024a). However, in previous works, the semantic versions of taxonomic descriptions were mostly vassals of natural language (NL) ones, the former being used to code phenotypes and the latter retained for human comprehension.

Here, we described a new species entirely using Phenoscript, a computer language specifically designed to make taxonomy computable. In contrast to previous works (Mikó et al. 2021, Montanaro et al. 2024a), we created semantic statements from the beginning, while observing specimens under the microscope and limited NL to comments guiding the descriptive process. Human readability is warranted by the NL file (markdown or html) generated in a matter of seconds from the Phenoscript file, which we supplied as a replacement for traditional descriptions. Our approach avoiding the creation of initial NL descriptions allows saving time and effort, indicating that Phenoscript can be a handy tool for taxonomists to create semantic descriptions and keeping good human intelligibility.

Lastly, we generated nanopublications to make the new taxonomic act and ecological data disclosed here readily available and searchable through the web, thus avoiding the need of data-mining into literature. Nanopubs are single, citable pieces of information that are becoming progressively important in the context of big data, since they make data easily accessible through the web (Kuhn et al. 2013, Kuhn and Dumontier 2017, Kuhn et al. 2021). They have already been used in dung beetle taxonomy to share synonymic and ecological information (Montanaro et al. 2024a, Rossini et al. 2024). Here, we further implemented them by testing a specific nanopub format for sharing new taxon names.

Overall, this article aims at providing a new standard for FAIR taxonomy papers able to face the call for semantic morphological data (Kuhn and Dumontier 2017, Yoder et al. 2018).

### The Malagasy Scarabaeus clade

Compared to the morphological homogeneity found in other Scarabaeini genera, Malagasy *Scarabaeus* show a relatively conspicuous degree of differentiation, which made their reciprocal relationships controversial (Paulian and Lebis 1960, Mostert and Holm 1982, Mostert and Scholtz 1986). Here, we can make the following observations supported by morphology and previous evidence:

1. *Scarabaeussakalava* sp. nov. is morphologically very close to *S.viettei* and thereby can be considered its sister species.
2. *Scarabaeussevoistra* shares with *S.sakalava* and *S.viettei* a very distinctive shape of male genitalia, specifically the ventral margin of parameres obtusely angled at half length and strongly concave before the distalmost end, which is bent ventrally (compare Fig. 4a, b with figs. 25–26 in Mostert and Holm (1982)). Genitalia are known to be particularly informative to infer phylogenetic relationships amongst dung beetles at tribal or lower taxonomic levels and might be taken as an important hint of relatedness between these species (Tarasov and Solodovnikov 2011, Génier and Moretto 2017). Additionally, the three species have an extremely similar cuticular sculpture and serrated ventral protibial margin in males. *S.sevoistra* also presents some highly derived characters (flightlessness, distally produced protibiae, adjacent mesocoxae etc.), which, however, are known to have evolved more than once in Scarabaeini following adaptation to arid environments (Mostert and Scholtz 1986, Harrison and Philips 2003, Harrison et al. 2003).
3. The most comprehensive molecular phylogeny of Scarabaeini found that *S.radama* and *S.viettei* form a clade nested within African *Scarabaeus* (Sole et al. 2011). The morphological similarities between *S.viettei*, *S.sakalava* and *S.sevoistra* suggest that *S.radama* is putatively close to the latter two as well.

Taken together, these observations provide good preliminary evidence that the Malagasy *Scarabaeus* species form a monophyletic group. According to the reconstruction by Sole et al. (2011), the clade diverged from its African relatives around 24–15 Mya, between late Oligocene and middle Miocene – long after the radiations of other Malagasy dung beetle clades, notably *Helictopleurus*, *Epilissus* and *Epactoides* and almost concurrently with the *Nanos* one (Wirta et al. 2008, Wirta et al. 2010).

With only four known species, Scarabaeini is the least diverse lineage amongst Malagasy dung beetles. This poor diversity is puzzling, also considering that the climate in southern Madagascar seems most suitable for this arid-adapted group. However, *Scarabaeus* radiation may have been hindered by two important factors: i) the limited amount of resources (especially wet dung of big mammals) available in the southern part of the island; ii) the competition with other dung beetle lineages that colonised the island long before Scarabaeini and effectively occupied available niches (Miraldo et al. 2011, Sole et al. 2011).

As to their higher-level systematics, Malagasy Scarabaeini are deeply nested within *Scarabaeus* in both morphological (Mostert and Scholtz 1986, Harrison and Philips 2003) and molecular (Sole et al. 2011) analyses. Specifically, the group appears to be sister to a clade formed by the genus *Escarabaeus* Zídek & Pokorný, 2011 and the species of *Scarabaeus* (*sensu lato*) close to *S.zambesianus*. At present, three solutions may be considered to deal with their systematics: 1) placing them in *Escarabaeus* after expanding the morphological concept of the genus defined by Ziani and Gudenzi (2012); 2) re-validating one or more genera in which the species were previously accommodated; 3) leaving them in *Scarabaeus* (*sensu lato*), pending a broader phylogenetic investigation. The third solution seems to be the most reasonable one at this stage. In fact, all the 11 currently recognised genera of Scarabaeini appear to be nested within *Scarabaeus*, interspersed with several species of *Scarabaeus* (*sensu lato*) (Harrison and Philips 2003, Forgie et al. 2005, Forgie et al. 2006, Sole et al. 2011). This troubled situation needs to be tackled in light of robust and fine-scale phylogenetic hypotheses and could end up in two alternative solutions: 1) splitting *Scarabaeus* in a plethora of genera or subgenera, several of which will probably be mono- or oligospecific; 2) lumping most or all currently recognised genera with *Scarabaeus* and, at most, subdividing the genus in informal species-groups. Whether to split or lump is quite subjective and we avoid discussing it here.

## Supplementary Material

XML Treatment for Scarabaeus sakalava

XML Treatment for Scarabaeus viettei

F47FF007-701D-5FDE-B649-46108E4AD326 10.3897/BDJ.12.e134364.suppl1 Supplementary material 1 Full data of Malagasy *Scarabaeus* specimens included in the study. Data type Excel table in Darwin Core Format File: oo_1145613.xlsx https://binary.pensoft.net/file/1145613 Giulio Montanaro

## Figures and Tables

**Table:** 

Nanopublication	Creator	Date
https://w3id.org/kpxl/pensoft/bdj/np/RANfU_7tS66XyfZS4RauqKLLnSEcfa1L06ueqKGLMU9TA	Giulio Montanaro	07-08-2024 10:04:55
https://w3id.org/kpxl/pensoft/bdj/np/RA2Vid3_DQcbuNapZTvi-ieGUnuZXk57hj3Itd-AIlscE	Giulio Montanaro	29-09-2024 17:48:48
https://w3id.org/kpxl/pensoft/bdj/np/RAcQ64ZIhNKjWvmPGhUaS4kOnUsaqtbgIr9Ln8ZlUbCmI	Giulio Montanaro	29-09-2024 18:12:37

**Figure 2a.**
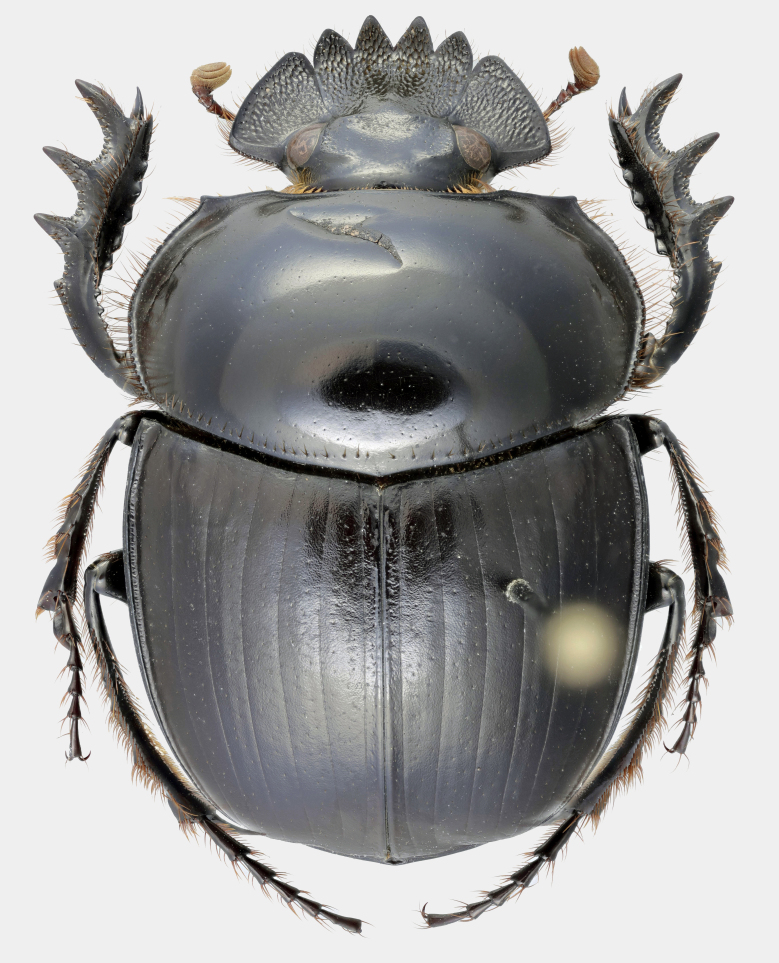
*S.sakalava* sp. nov., male (holotype);

**Figure 2b.**
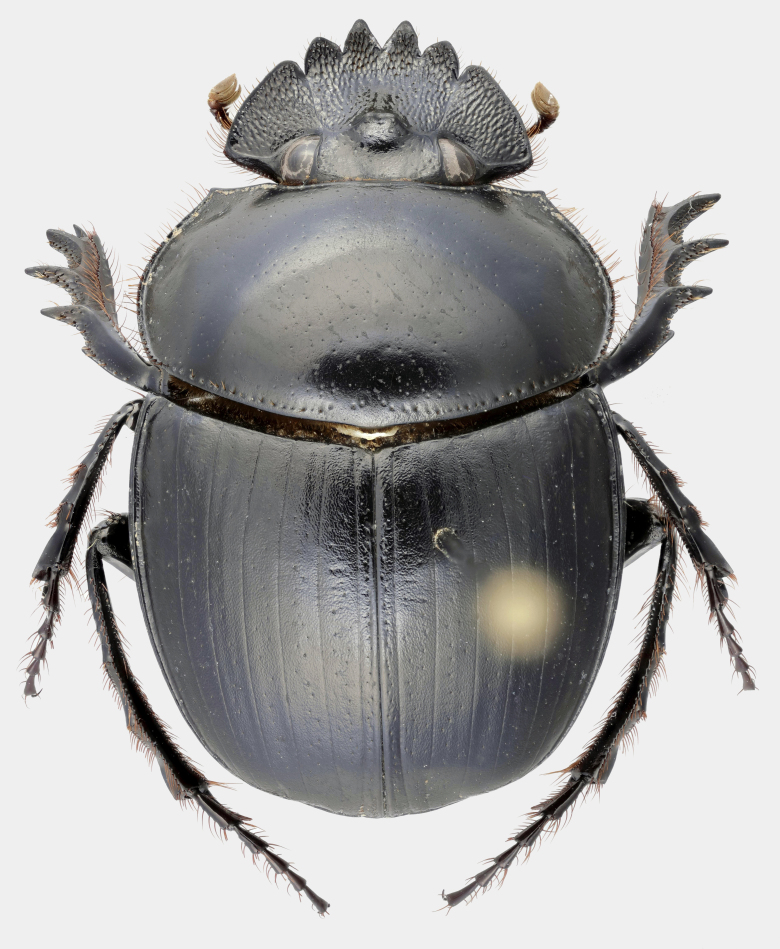
*S.sakalava*, female (paratype);

**Figure 2c.**
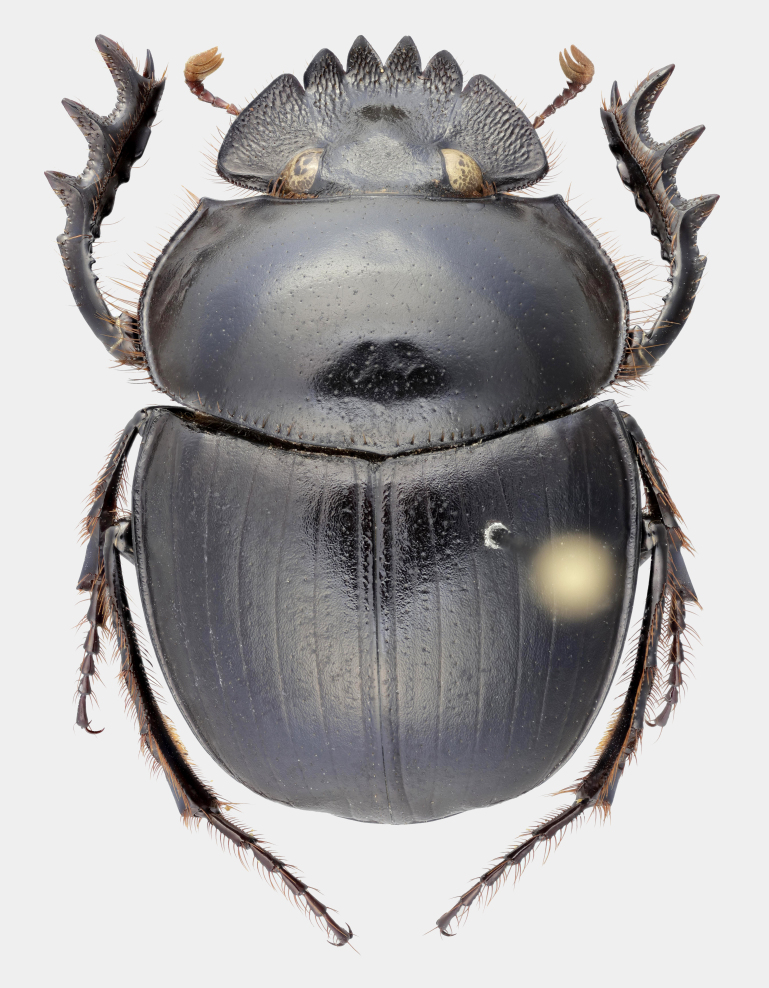
*S.viettei* (Paulian, 1953), male;

**Figure 2d.**
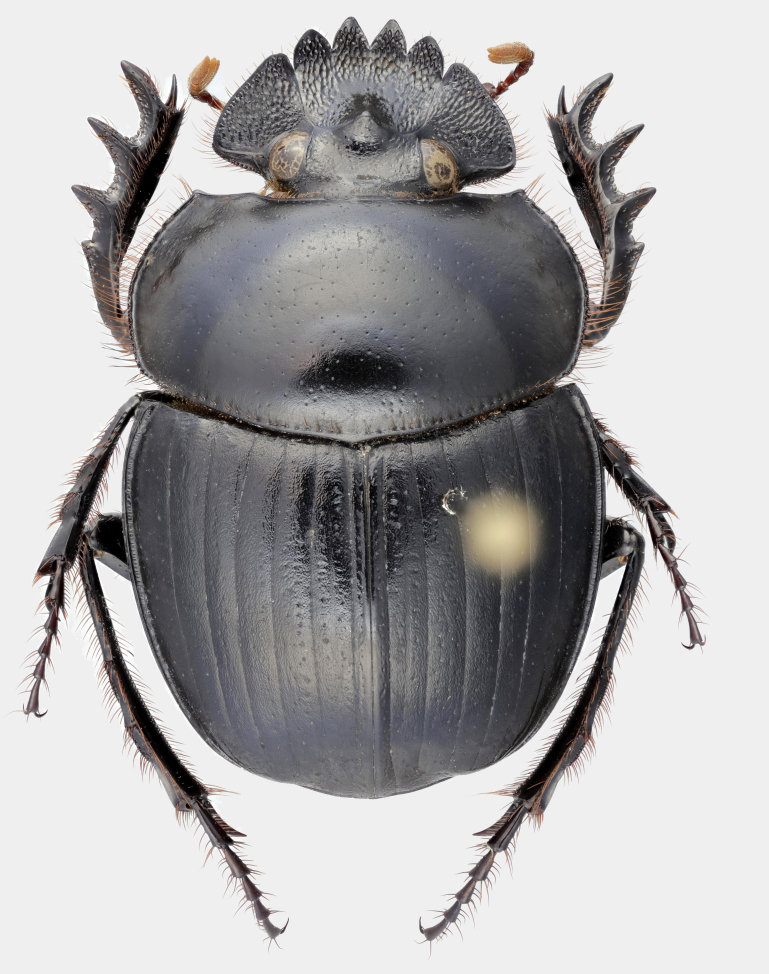
*S.viettei*, female.

**Figure 3a.**
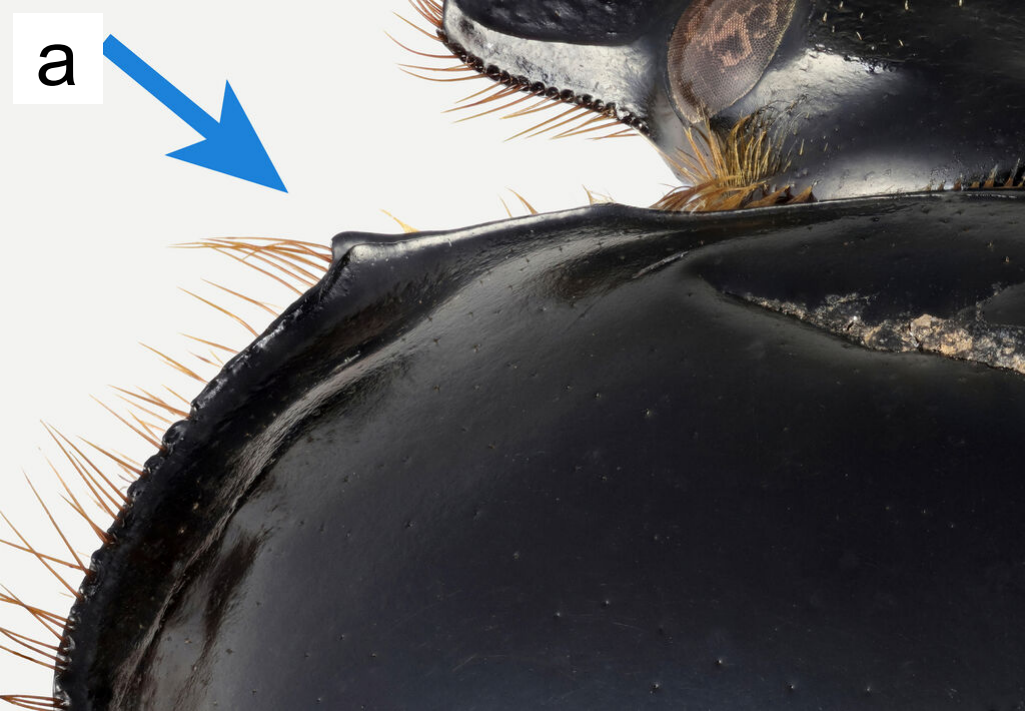
*S.sakalava* sp. nov. (holotype), anterolateral pronotal angle;

**Figure 3b.**
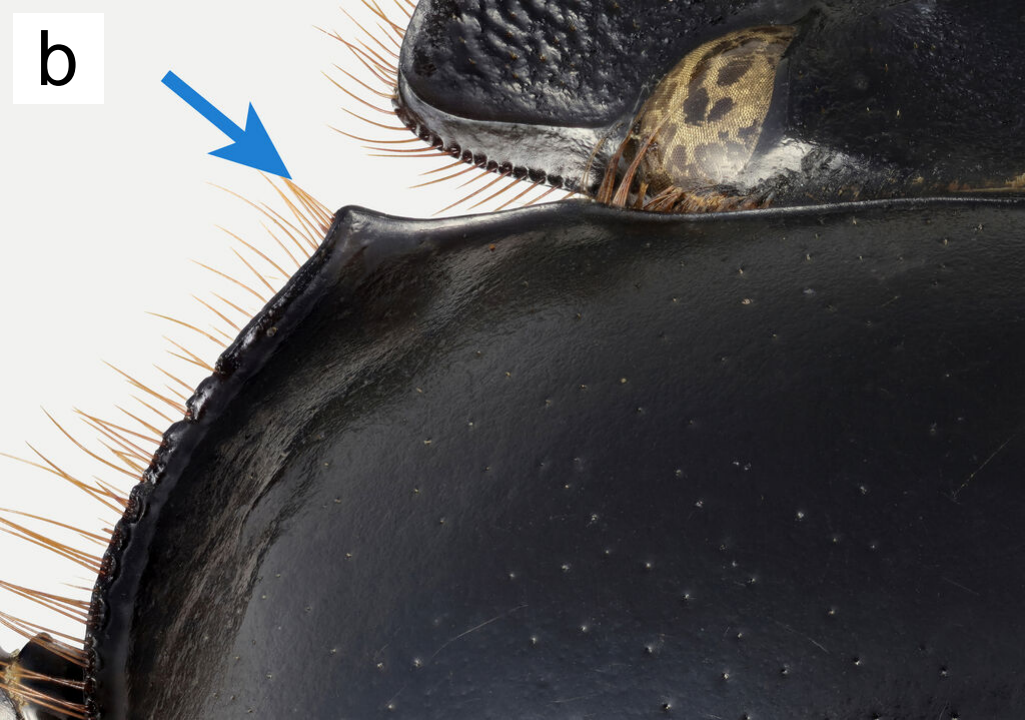
*S.viettei* (Paulian, 1953), anterolateral pronotal angle;

**Figure 3c.**
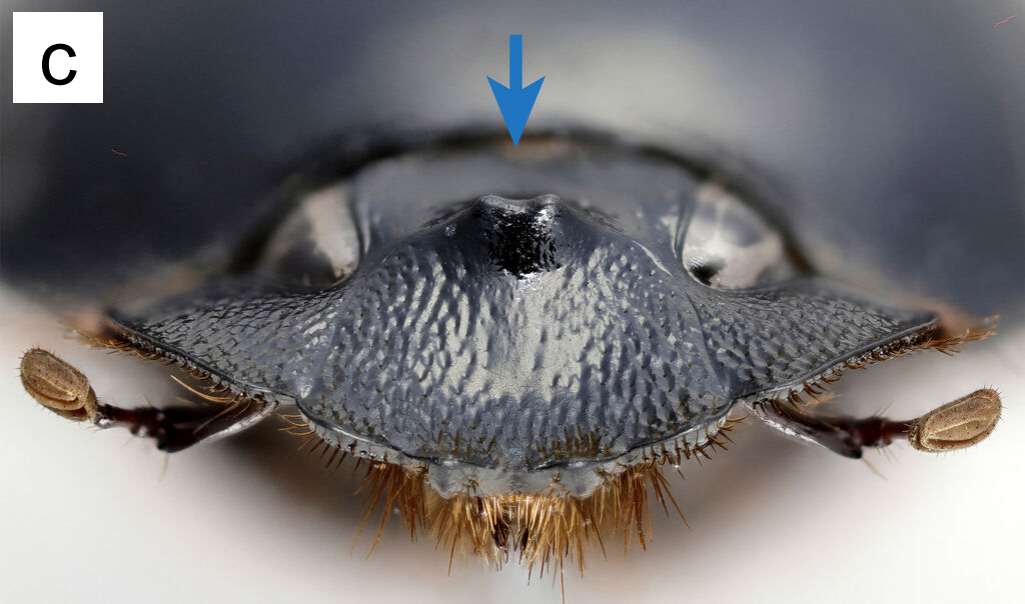
*S.sakalava* (paratype), female frontal tubercle;

**Figure 3d.**
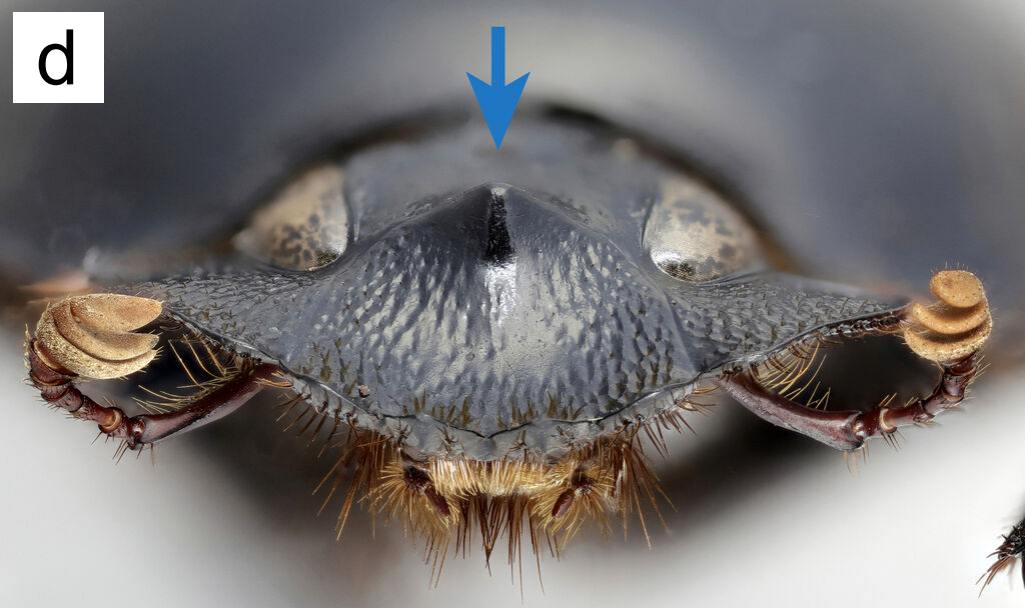
*S.viettei*, female frontal tubercle;

**Figure 3e.**
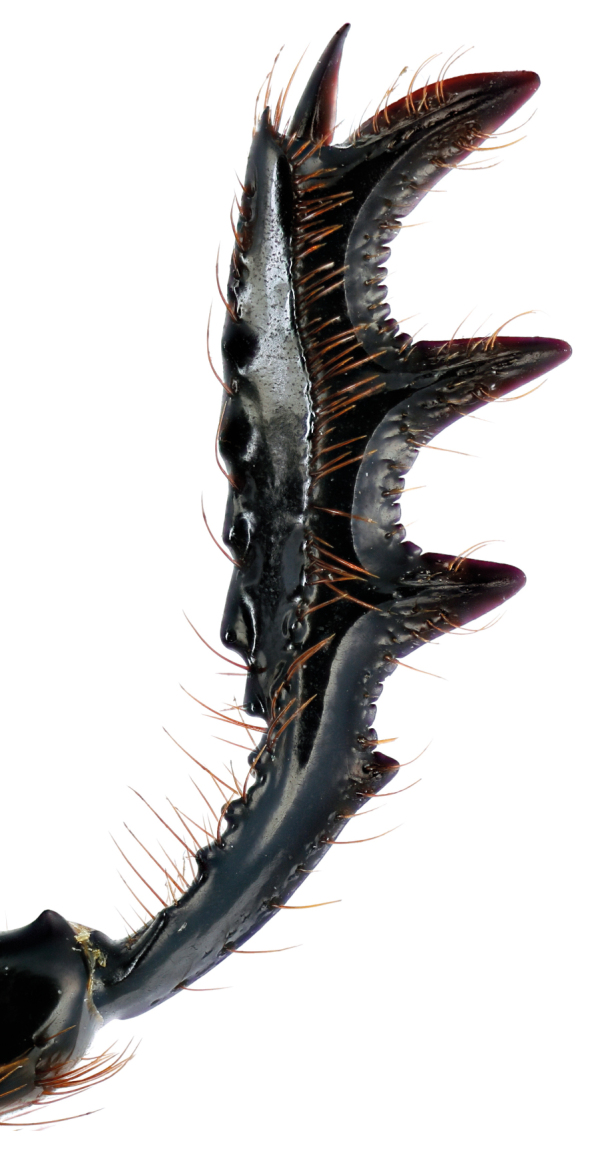
*S.sakalava* (holotype), male protibia;

**Figure 3f.**
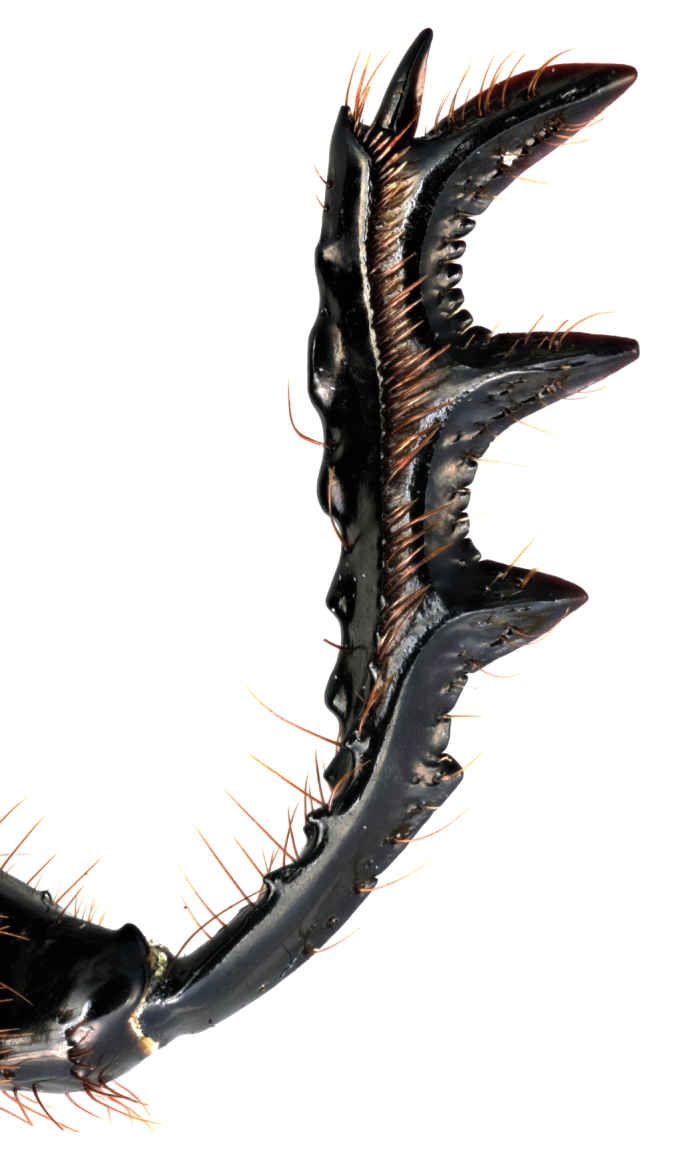
*S.viettei*, male protibia.

**Figure 4a.**
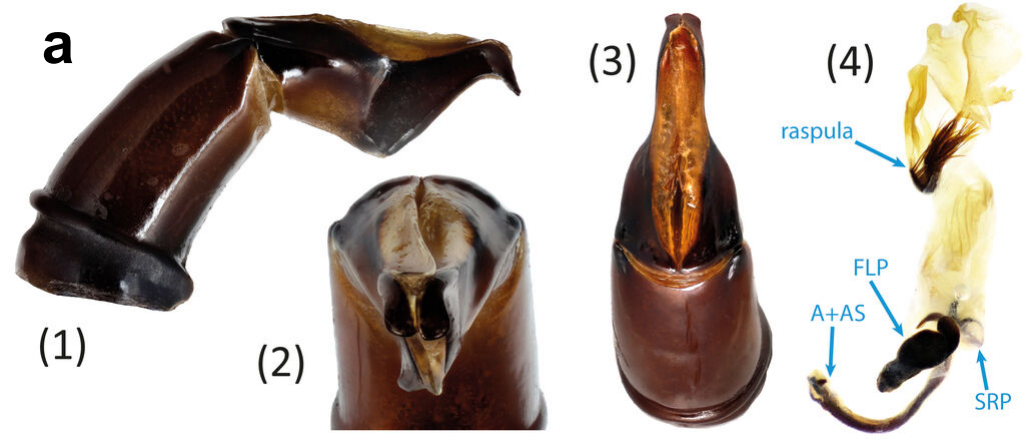
*S.sakalava* sp. nov.;

**Figure 4b.**
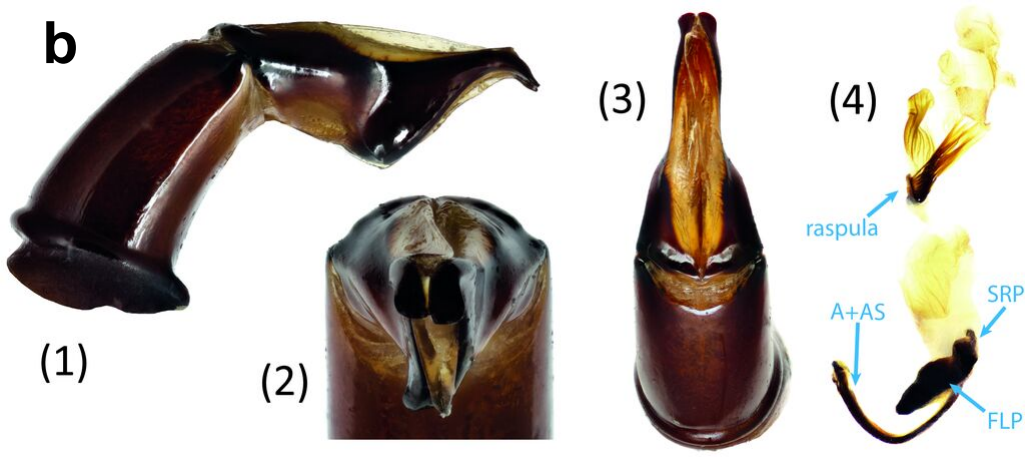
*S.viettei* (Paulian, 1953);

**Figure 4c.**
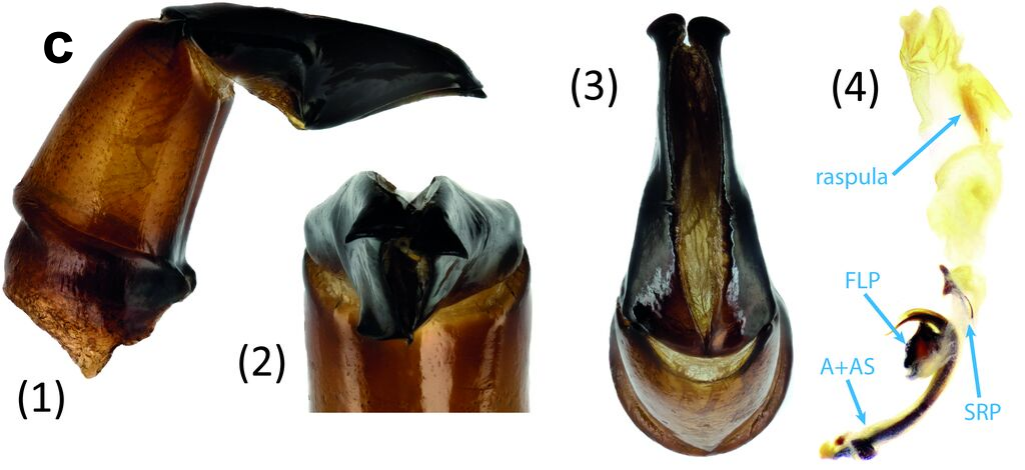
*S.radama* Fairmaire, 1895.
